# *Taenia solium* Human Cysticercosis: A Systematic Review of Sero-epidemiological Data from Endemic Zones around the World

**DOI:** 10.1371/journal.pntd.0003919

**Published:** 2015-07-06

**Authors:** Marco Coral-Almeida, Sarah Gabriël, Emmanuel Nji Abatih, Nicolas Praet, Washington Benitez, Pierre Dorny

**Affiliations:** 1 Institute of Tropical Medicine, Department of Biomedical Sciences, Antwerp, Belgium; 2 Ghent University, Faculty of Veterinary Medicine, Merelbeke, Belgium; 3 Universidad Central del Ecuador, Centro Internacional de Zoonosis (CIZ), Ciudadela Universitaria, Quito, Ecuador; 4 Universidad de las Américas, Escuela de Medicina Veterinaria y Zootecnia, Quito, Ecuador; 5 Universidad Central del Ecuador, Facultad de Medicina Veterinaria y Zootecnia, Ciudadela Universitaria, Quito, Ecuador; University of Zurich, SWITZERLAND

## Abstract

**Background:**

*Taenia solium* cysticercosis is a zoonotic neglected disease responsible for severe health disorders such as seizures and death. Understanding the epidemiology of human cysticercosis (HCC) in endemic regions will help to expose critical information about the transmission of the disease, which could be used to design efficient control programs. This review gathered serological data on apparent prevalence of *T*. *solium* circulating antigens and/or seroprevalence of *T*. *solium* antibodies, apparent prevalence of human taeniasis and risk factors for HCC from endemic communities in order to understand the differences in exposure to the parasite and active infections with *T*. *solium* metacestodes in endemic areas around the world.

**Methods:**

Three databases were used to search sero-epidemiological data from community-based studies conducted between 1989 and 2014 in cysticercosis endemic communities worldwide. The search focused on data obtained from *T*. *solium* circulating antigen detection by monoclonal antibody-based sandwich ELISA and/or *T*. *solium* antibody seroprevalence determined by Enzyme-linked Immunoelectrotransfer Blot (EITB). A meta-analysis was performed per continent.

**Principal Findings:**

A total of 39,271 participants from 19 countries, described in 37 articles were studied. The estimates for the prevalence of circulating *T*. *solium* antigens for Africa, Latin America and Asia were: 7.30% (95% CI [4.23–12.31]), 4.08% (95% CI [2.77–5.95]) and 3.98% (95% CI [2.81–5.61]), respectively. Seroprevalence estimates of *T*. *solium* antibodies were 17.37% (95% CI [3.33–56.20]), 13.03% (95% CI [9.95–16.88]) and 15.68% (95% CI [10.25–23.24]) respectively. Taeniasis reported prevalences ranged from 0 (95% CI [0.00–1.62]) to 17.25% (95% CI [14.55–20.23]).

**Significance:**

A significant variation in the sero-epidemiological data was observed within each continent, with African countries reporting the highest apparent prevalences of active infections. Intrinsic factors in the human host such as age and immunity were main determinants for the occurrence of infections, while exposure was mostly related to environmental factors which varied from community to community.

## Author Summary

Human cysticercosis is a neglected zoonotic disease caused by the larval stage of the parasite *Taenia solium*. This disease is responsible for severe health disorders such as seizures, and in some cases even death. *T*. *solium* cysticercosis is endemic in countries in Africa, Latin America and Asia where conditions such as inadequate hygiene, poor sanitary conditions, open defecation, free roaming pigs and poverty permit the transmission of the disease. Diagnostic tools are capable of detecting exposure to eggs and infection levels in a population through antibody and antigen detection, respectively. This review focused on gathering epidemiological data from endemic communities in Africa, Latin America and Asia in order to understand the causes of the observed variations in exposure/infection patterns in endemic regions. Similar antibody seroprevalences were observed worldwide while infection prevalences varied significantly within each region. Intrinsic factors such as age and immunity were determining factors for the occurrence of infections, while exposure was related to environmental factors which varied from region to region. Understanding the epidemiology of cysticercosis in endemic regions will help expose information on the transmission, which could in turn be used to design appropriate control programs.

## Introduction

*Taenia solium* human cysticercosis (HCC) is a zoonotic parasitic disease causing severe health and economic problems in endemic areas in Latin America, Africa and Asia [1–4]. The disease is related to poor sanitary conditions, inadequate hygiene, open defecation, presence of free roaming pigs and poverty [5;6]. The natural life cycle of *T*. *solium* includes humans as the only definitive hosts carrying the intestinal adult tapeworm, and pigs as the intermediate hosts infected with the metacestode larval stage (cysticercus), generally in the muscular tissue. Humans acquire a *T*. *solium* tapeworm infection (taeniasis) by consumption of undercooked pork containing viable cysticerci. Pigs contract porcine cysticercosis (PCC) by ingestion of viable *T*. *solium* eggs contained in feces from human tapeworm carriers. HCC occurs when humans accidentally ingest *T*. *solium* eggs and develop the larval stage of *T*. *solium* in different tissues. Once established in the tissue of the intermediate host, the cysticercus develops into the viable stage, which is composed of a scolex visible through vesicular fluid and an opaline membrane inside a cyst [7]. After a few months or years, depending on the host immune response, the cysticercus starts degenerating, the vesicular fluid becomes dense and opaque, the cyst loses also its regular shape and becomes smaller. Finally, the cysticercus undergoes the stage of calcification in which it ends as a round white calcified nodule [8]. Neurocysticercosis (NCC) occurs when the larval stage establishes in the central nervous system [9]. NCC is the most severe presentation of the infection and is considered the most important parasitic disease of the neural system, being responsible for almost one third of the acquired epilepsy cases in endemic areas [2]. HCC can also involve muscular and ocular disorders, infection of subcutaneous tissue [10–12] and in severe cases can even cause death [13]. Even though HCC is considered potentially eradicable, it is still highly prevalent in developing countries [14]. Different intervention measures have to be integrated to interrupt transmission of *T*. *solium*. Effective control programs will reduce the incidence and prevalence of the disease leading to acceptable and manageable levels, which could therefore become a first step towards elimination and eradication of the disease. Understanding the conditions for transmission is required for developing appropriate interventions [15]. This mainly involves accurate estimations of exposure and infection patterns in communities, which can be obtained by laboratory tests [15;16]. Current immunological tools used in HCC diagnosis can be classified into: 1) Tests detecting antibodies directed against *T*. *solium* cysticerci, identifying exposure to the parasite, and 2) Tests detecting circulating antigens produced by living cysticerci, identifying current infection with viable cysticerci [17]. Measuring the level of adult tapeworm infections in a population can be used as a support for results obtained from immunological tests to measure exposure. To understand exposure and infection patterns it is also important to have a correct interpretation of risk factors, information that can be provided by studying the correlation between host, environment and parasite factors and serological results for cysticercosis.

Similar sero-epidemiological studies conducted in two endemic communities in Africa and Latin America, presented comparable serological results for exposure to *T*. *solium* eggs with exposure levels of 34.55% and 31.22%, respectively but presented significant differences when reporting active infections with almost 12 times more infections in the African than in the Latin American community [16;18], suggesting that there were significant variations in the conditions for transmission and in the establishment of infection in each community. For this reason, extrapolating results from a single community to a regional or even global level can be a hazardous exercise.

The aim of this review is to systematically collect serological data on apparent prevalence of *T*. *solium* circulating antigens and/or seroprevalence of *T*. *solium* antibodies, apparent prevalence of human taeniasis and risk factors for HCC from endemic communities in order to understand the differences in exposure to the parasite and active infections with *T*. *solium* metacestodes in endemic areas around the world.

## Methods

A systematic literature search on *T*. *solium* HCC seroprevalence in community-based studies performed in endemic countries in Africa, Asia, Latin America and the Caribbean was conducted on indexed literature published during the period from 1989 to 2014. In order to have comparable data, this search focused on the articles in which data was obtained using the following techniques and protocols: 1) Enzyme-linked Immunoelectrotransfer Blot (EITB) from Tsang et al. (1989) [19] for detection of antibodies directed against seven specific glycoproteins from *T*. *solium* metacestodes and/or 2) Enzyme Linked Immunosorbent Assay detecting circulating antigens from the *T*. *solium* metacestode (B158/B60 Ag-ELISA or HP10 Ag-ELISA) from Brandt et al. (1992) [20], Van Kerckhoven et al. (1998) [21], Dorny et al. (2000) [22] and Harrison et al. (1989) [23]. Selection was restricted to both EITB and Ag-ELISA because of their performance, frequent use and acceptance as highly sensitive and specific tests in community-based studies when compared to other techniques. The EITB has a sensitivity ranging from 97 to 98% and a specificity ranging from 97 to 100% to detect circulating antibodies to *T*. *solium* in human serum. The EITB is considered positive when at least one of the seven specific glycoproteins from *T*. *solium* metacestodes is recognized by the serum The B158/B60 Ag-ELISA has a sensitivity of 90% (95% CI:[80–99%]) and a specificity of 98% (95% CI:[97–99%]) to detect circulating antigens released by *T*. *solium* metacestodes in humans, with no cross-reactions reported to date [17;19;24]. For the HP10 Ag-ELISA the reported sensitivity ranges from 84.8% to 86% and the specificity is estimated at 94% to detect circulating antigens in human serum [25;26]. For the studies where the EITB was performed, either the Centers for Disease Control and Prevention (CDC) test or commercial kits were accepted for selection. Taeniasis apparent prevalence data was collected when available in the selected articles and no restrictions for the diagnostic method was made. It was anticipated that the taeniasis data would be influenced by the technique used because coprology, coproantigen ELISA and molecular methods have wide differences in their diagnostic performances. The estimated sensitivities of these tests were: 52.5% (95% CI:[11.1–96.5]) for coprology, 84.5% (95% CI:[61.9–98]) for coproantigen ELISA and 82.7% (95% CI:[57–97.6]) for real-time polymerase chain reaction assay (copro-PCR) and their specificities were: 99.9% (95% CI:[99.5–100]) for coprology, 92% (95% CI: [90–93.8]) for coproantigen ELISA and 99% (95% CI:[98.2–99.6]) for copro-PCR [27]. The wide confidence interval for the estimated value for the sensitivity of coprology is due to the low number of positive cases found by this method, which does not allow a more accurate estimation, however is in line with previous studies [28–30]. Language restriction was applied when the article was written in a language other than those spoken or understood by the authors of this review. The considered languages were English, Spanish, French, Portuguese and Dutch.

The selected databases for this study were: PubMed (http://www.ncbi.nlm.nih.gov/pubmed/), LILACS (Latin American and Caribbean Health Sciences Literature, lilacs.bvsalud.org/en/) and Web Of Science (http://wok.mimas.ac.uk/). The search was performed from September 1^st^ 2013 until October 31^st^ 2014.

### Search

The following search strategy was applied: In PubMed, using the Boolean operator AND, the terms “cysticercosis” AND “*Taenia solium*” AND “epidemiology” were introduced in the main search bar and the filters were activated for the period from 1988/12/31 to 2014/10/31. In Web of Science, the strategy applied was introducing in the basic search bar the topic “cysticercosis” adding fields with the correspondent Boolean operators: AND Topic = (*Taenia solium*) AND Topic = (epidemiology). In LILACS, the strategy adopted consisted in introducing in the main search bar the terms “cysticercosis *Taenia solium*”. In the latter case the term “epidemiology” was excluded to obtain the maximum return of articles since LILACS is a smaller targeted database when compared to PubMed and Web Of Science. Additionally, relevant articles were included that were not found with this search strategy but matched the selection criteria after manual search or expert recommendation.

### Study selection

The articles were selected following three phases: The first phase consisted in the removal of all repeated studies from the title selection and all studies performed before 1989. The second phase consisted in the exclusion of articles from the title and abstract review as for the following exclusion criteria: 1) Wrong parasite species, 2) Studies performed in non-endemic countries, 3) Studies performed only in animals, 4) Clinical studies, 5) Studies which focused only on human taeniasis, 6) Studies carried out for assessing laboratory tests performance, 7) Studies that focused on NCC, 8) Studies conducted in specific targeted types of individuals (e.g. schoolchildren, refugees or soldiers), 9) Articles written in languages other than those spoken or understood by the authors of this review, 10) Interviews, letters, reviews or editorials not presenting original data and/or the techniques and protocols performed on their studies, 11) Studies not related to *T*. *solium* epidemiology. The third phase was applied when full texts were read and consisted in the study selection according to the following selection criteria: 1) Community-based studies, 2) Original HCC prevalence reports available, 3) Protocols applied for HCC diagnosis using the EITB and Ag-ELISA protocols mentioned previously in this document, 4) Random sampling method for selection of participants in the study or/and population representative voluntary participation. 5) Coverage of most age groups (young, adults and elderly).

### Data collection

From every selected article the following items were collected and introduced in a data base: Author(s), year the article was published, country, number of participants for Ag-ELISA survey, number of Ag-ELISA positive detected cases, number of participants for EITB survey, number of EITB positive detected cases (when specified in the article, the EITB positive cases were considered when at least one band had a visible reaction [19]), estimated apparent prevalence expressed in percentage. When applicable, identified risk factors were also included in the database. For longitudinal studies where two or more sampling rounds were organized in a community, only data from the first round were taken into account in order to avoid any possible bias caused after contact with the teams collecting the samples. Additionally, if the study was conducted in parallel with a survey on human taeniasis, the prevalence and the method used to diagnose taeniasis were also gathered. All countries where the selected studies took place were visualized using QGIS software (version2.8).

### Statistical analysis

A meta-analysis was conducted using the “meta” package in R [31] to estimate the prevalence of circulating antigens for *T*. *solium* and the seroprevalence of antibodies in each continent based on a random effects model. A global prevalence estimation was not performed as it was not the intention of this study. Ninety five % exact binomial Confidence Intervals (95% CI) were calculated for every reported prevalence. The significant differences in estimated prevalences were evaluated using their 95% confidence intervals. The difference in prevalence between two regions was considered to be statistically significant if their 95% confidence intervals do not overlap.

## Results

### Study selection

**Fig 1:**
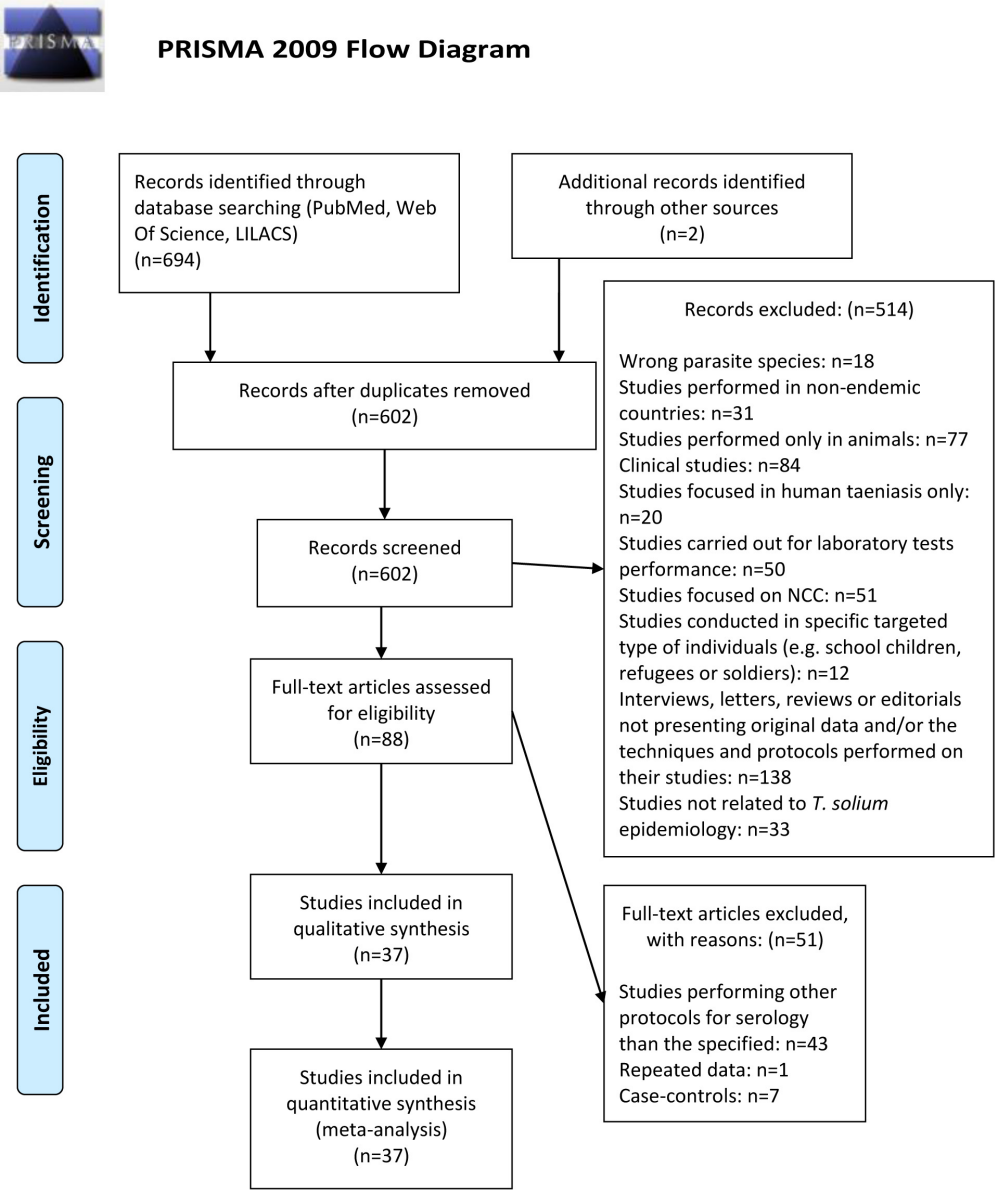
Flow diagram describing literature search and selection of studies (PRISMA 2009 flow chart).

Fig 1 describes the review process and the number of articles selected at each stage of the review. From an initial number of 696 articles, only 37 studies were included in this review; 9 studies were selected for the African region [18;32–39], 22 studies for Latin America [16;40–60] and 6 for the Asian region.

### Human cysticercosis in Africa

From the 9 sero-epidemiological studies from 7 countries selected for the African region, 7 studies [32–34;36–39] used Ag-ELISA and two studies [18;35] used both Ag-ELISA and EITB. The total number of individuals sampled for serological testing in this region was 12,596. Prevalence of circulating antigens ranged from 0.68% to 21.63%, while seroprevalence of antibodies ranged from 7.69 to 34.55%. Detailed descriptions of each study are given in Table 1.

**Table 1:** Sero-epidemiological studies using Ag-ELISA and/or EITB for human cysticercosis in Africa.

Author	YOP	Country	Location	Circulating Ag positive cases/total participants	Circulating Ag prevalence (%) [95%CI]	Ab positive cases/total participants	Ab seroprevalence (%) [95%CI]
Nguekam et al.	2003	Cameroon	West province	34^a^/4993	0.68 [0.47–0.95]		
Carabin et al.	2009	Burkina Faso	Sanguié, Kadogo, Oubritenga	48^a^/763	6.29 [4.67–8.25]		
Kanobana et al.	2011	Dem. Rep. of Congo	Bas-Congo	204^a^/943	21.63 [19–24.4]		
Secka et al.	2011	Senegal	Ziguinchor	31^a^/403	7.69 [5.28–10.74]	31/403	7.69 [5.28–10.74]
Nitiéma et al.	2012	Burkina Faso	Sanguié, Kadogo, Oubritenga	45^a^/734	6.13 [4.50–8.12]		
Mwape et al.	2012	Zambia	Estern Province	41^a^/707	5.80 [4.19–7.8]		
Mwape et al.	2013	Zambia	Eastern Province	141^a^/1129	12.49 [10.61–14.56]	57/165	34.55 [27.32–47.33]
Mwanjali et al.	2013	Tanzania	Mbeya	139^a^/830	16.75 [14.27–19.46]		
Thomas	2014	Kenya	Western and Nyanza	169^b^/2094	8.07 [6.94–9.32]		
**Total**				**852/12596**	**6.76 [6.33–7.22]**	**88/568**	**15.49 [12.61–18.73]**
**REMEP:**					**7.30 [4.23–12.31]**		**17.37 [3.33–56.20]**

Legend: YOP: Year of publication; Location: Corresponds to the Province, State, Region or Department in which the communities where the studies took place are located; Ag: Antigen detection based on Ag-ELISA results; Ab: Antibody detection based on EITB results; 95% CI: 95% Confidence Intervals; REMEP: Random Effects Model Estimated Prevalence; Dem. Rep. of Congo: Democratic Republic of Congo.

^a^: Results obtained from B158/B60 Ag-ELISA

^b^: Results obtained from HP10 Ag-ELISA.

### Human cysticercosis in Latin America

Information on HCC in Latin America was obtained from 22 studies from 8 countries with two studies performing only Ag-ELISA [44;55], 17 studies performing EITB only [40–43;45–54;57–59] and three studies performing both Ag-ELISA and EITB [16;56;60]. The total number of individuals sampled was 21,911. Active *T*. *solium* infection ranged from 0.94 to 9.12% and antibody seroprevalence ranged from 1.82 to 31.22%. A detailed description of each study is given in Table 2.

**Table 2:** Sero-epidemiological studies using Ag-ELISA and/or EITB for human cysticercosis in Latin America.

Author	YOP	Country	Location	Circulating Ag positive cases/Total participants	Circulating Ag Prevalence (%) [95%CI]	Ab positive cases/Total participants	Ab seroprevalence (%) [95%CI]
Díaz et al.	1992	Peru	San Martin			30/371	8.09 [5.52–11.34]
Sarti et al.	1992	Mexico	Morelos			167/1546	10.80 [9.3–12.46]
Sarti et al.	1994	Mexico	Michoacan			49/1005	4.88 [3.63–6.39]
García-Noval et al.	1996	Guatemala	Jutiapa			207/1542	13.42 [11.76–15.23]
Sanchez et al.	1998	Honduras	Francisco Morazán			63/404	15.59 [12.2–19.5]
García et al.	1998	Peru	Cusco			24/102	23.8 [15.69–32.96]
Sanchez et al.	1999	Honduras	Olancho			80/480	16.67 [13.44–20.31]
García et al.	1999	Peru	Cusco			14/108	12.96 [7.27–20.8]
Sarti et al.	2000	Mexico	Morelos			91/1603	5.68 [4.59–6.92]
García et al.	2001	Peru	Piura			76/482	15.77 [12.63–19.33]
		Peru	Junín			140/398	35.18 [30.48–40.1]
		Colombia	N.A.			23/750	3.07 [1.95–4.57]
Gomes et al.	2002	Brazil	Bahia			11/694	1.59 [0.79–2.82]
García et al.	2003	Peru	Junín			355/2583	13.74 [12.44–15.13]
Ferrer et al.	2003	Venezuela	Carabobo	27^b^/296	9.12 [6.10–12.99]		
		Venezuela	Lara	37^b^/608	6.08 [4.32–8.29]		
		Venezuela	Lara	20^b^/305	6.56 [4.05–9.95]		
Agudelo-Flores & Palacio	2003	Colombia	Antioquia			12/661	1.82 [0.94–3.15]
Moro et al.	2003	Peru	Lima			66/316	20.89 [16.54–25.79]
Rodriguez et al.	2003	Ecuador	Pichincha & Imbabura	215^a^/4306	4.99 [4.36–5.69]		
Montano et al.	2005	Peru	Tumbes			200/825	24.24 [21.35–27.32]
Rodriguez et al.	2006	Ecuador	Loja	18^a^/800	2.25 [1.34–3.53]	40/100	40.00 [30.33–50.23]
Agudelo-Flores et al.	2009	Colombia	Chocó			4/46	8.70 [2.42–20.79]
Lescano et al.	2009	Peru	Tumbes			196/803	24.41 [21.47–27.53]
Praet et al.	2010	Ecuador	Loja	23^a^/794	2.90 [1.84–4.31]	202/807	25.03 [22.08–28.17]
Coral et al.	2014	Ecuador	Loja	7^a^/744	0.94 [0.38–1.93]	232/743	31.22 [27.9–34.69]
**Total**				**347/7853**	**4.42 [3.97–4.90]**	**2282/16369**	**13.94 [13.41–14.48]**
**REMEP:**					**4.08 [2.77–5.95]**		**13.03 [9.95–16.88]**

Legend: YOP: Year of publication; Location: Corresponds to the Province, State, Region or Department in which the communities are located where the studies took place; Ag: Antigen detection based on Ag-ELISA results; Ab: Antibody detection based on EITB results; 95% CI: 95% Confidence Intervals; REMEP: Random Effects Model Estimated Prevalence

^a^: Results obtained from B158/B60 Ag-ELISA

^b^: Results obtained from HP10 Ag-ELISA

N.A.: Not available.

### Human cysticercosis in Asia

The data obtained for HCC in Asia resulted from 6 studies from 4 countries organized in 4 studies performing Ag-ELISA only [24;61–63], one study performing EITB only [64] and one study performing both Ag-ELISA and EITB [65]. The total number of individuals sampled was 4,764. Seroprevalence varied from 0.57 to 5.71% for circulating *T*. *solium* metacestode antigens and from 12.60 to 19.17% for *T*. *solium* antibody seroprevalence. Detailed descriptions of each study are given in Table 3.

**Table 3:** Sero-epidemiological studies using Ag-ELISA and/or EITB for human cysticercosis in Asia.

Author	YOP	Country	Location	Circulating Ag positive cases/total participants	Circulating Ag prevalence (%) [95%CI]	Ab positive cases/total participants	Ab seroprevalence (%) [95%CI]
Theis et al.	1994	Indonesia	Bali			94/746	12.60 [10.3–15.2]
Erhart et al.	2002	Viet Nam	Bac Ninh	12^a^/210	5.71 [2.99–9.77]		
Somers et al.	2006	Viet Nam	Bac Kan	16^a^/303	5.28 [3.05–8.43]		
		Viet Nam	Ha Tinh	1^a^/175	0.57 [0.01–3.14]		
Raghava et al.	2010	India	Tamil Nadu	46^a^/960	4.79 [3.53–6.34]	184/960	19.17 [16.72–21.8]
Jayaraman et al.	2011	India	Tamil Nadu	48^a^/1064	4.51 [3.34–5.94]		
Conlan et al.	2012	Laos	Oudomxay, Luangprabang, Huaphan, and Xiengkhuang	29^a^/1306	2.22 [1.49–3.17]		
**Total**				**152/4018**	**3.78 [3.21–4.42]**	**278/1706**	**16.29 [14.57–18.13]**
**REMEP**					**3.98 [2.81–5.61]**		**15.68 [10.25–23.24]**

Legend: YOP: Year of publication; Location: Corresponds to the Province, State, Region or Department in which the communities are located where the studies took place; Ag: Antigen detection based on Ag-ELISA results; Ab: Antibody detection based on EITB results; 95% CI: 95% Confidence Intervals; REMEP: Random Effects Model Estimated Prevalence

^a^: Results obtained from B158/B60 Ag-ELISA

**Fig 2:**
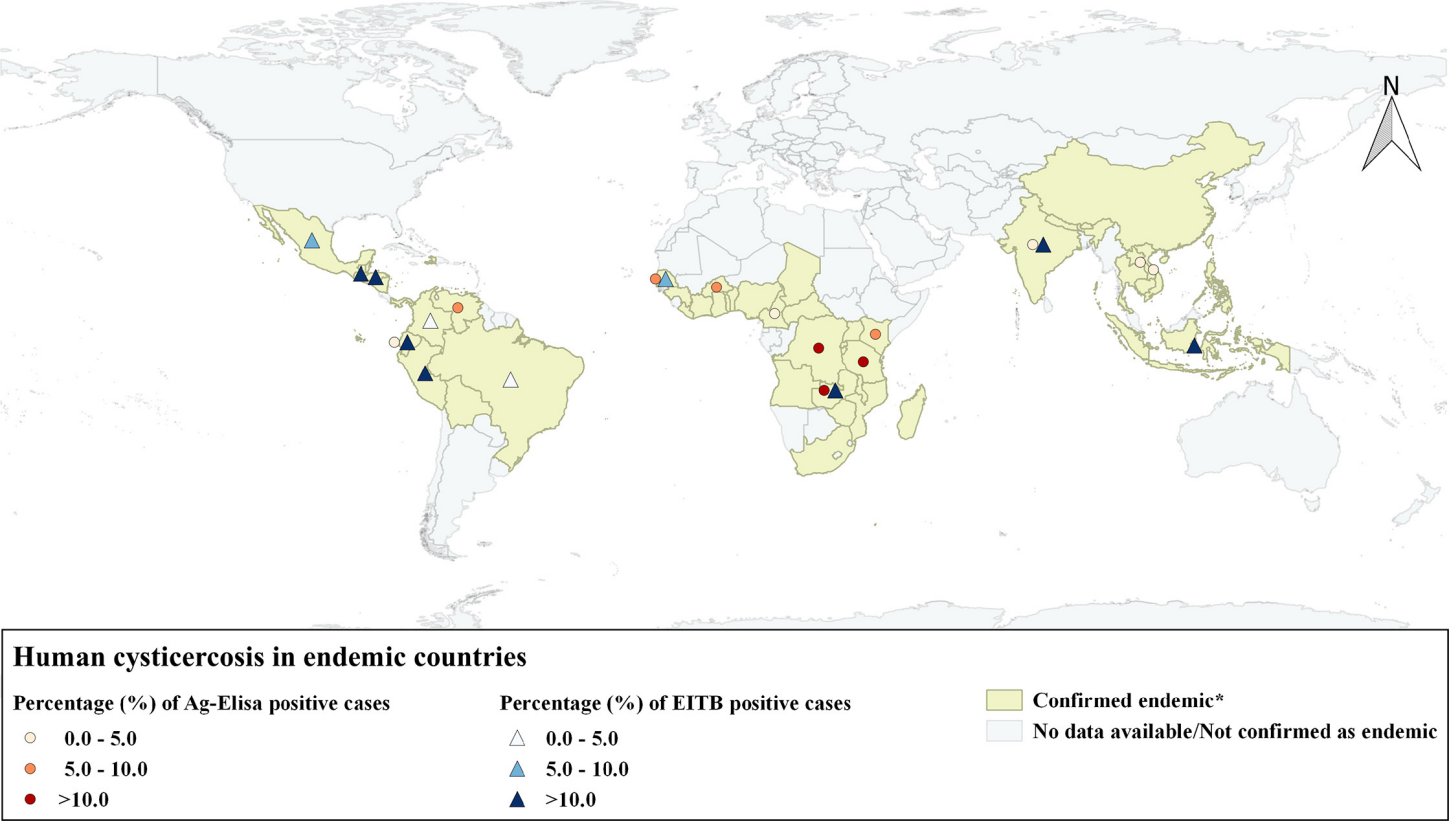
Global distribution of the endemic countries where Ag-ELISA and/or EITB based epidemiological studies were held. Light yellow represents the countries confirmed as endemic by the World Health Organization (WHO) until 2012 [66]. Circles represent the countries where Ag-ELISA based studies took place. Each color represents the average prevalence per country found from the selected articles in this review classified in 0 to 5 percent; 5 to 10 percent and more than 10 percent. Triangles represent the countries where EITB based studies took place. Each color represents the average prevalence per country found from the selected articles in this review classified in 0 to 5 percent; 5 to 10 percent and more than 10 percent.

Globally, 39,271 participants from 19 countries were included in 37 studies, from which 24,467 were studied for circulating *T*. *solium* antigen with 5.52% (1,351/24,467) positive cases (95% CI [5.24–5.82]) and 18,643 were studied for anti *T*. *solium* antibodies with a crude seroprevalence of 14.20% (2,648/18,643) (95% CI [13.70–14.71]). Fig 2 represents the global distribution of the countries where the serological studies took place.

### Taeniasis

Within the examined reports, twenty-four studies from 14 countries in Africa (6 studies), Latin America (13 studies) and Asia (5 studies) reported adult *T*. *solium* data, using different diagnostic techniques. In these studies, 4,682 African, 13,782 Latin American and 3,437 Asian subjects participated; with a global participation of 21,901 individuals. Apparent prevalence on taeniasis varied from 0 to 17.25%. Results are shown in Table 4.

**Table 4:** Taeniasis reports from Africa, Latin America and Asia.

Continent	Author	Year of publication	Country	Diagnostic Technique	Positive cases/ Total participants^a^	Prevalence (%) [95%CI]
Africa	Kanobana et al.	2011	Dem. Rep. of Congo	Coprology†	3/816	0.37 [0.08–1.07]
	Secka et al.	2011	Senegal	Direct fecal examination	2/43**	4.65 [0.57–15.81]
				FECT	4/43**	9.30 [2.59–22.14]
				worm expulsion and morphological identification	1/43**	2.33 [0.06–12.29]
	Mwape et al.	2012	Zambia	FECT	2/718	0.28 [0.03–1.00]
				Copro-Ag ELISA	45/712	6.32 [4.65–8.37]
	Mwanjali et al.	2013	Tanzania	Copro-Ag ELISA	43/820	5.24 [3.82–7.00]
				EITB (rES38)	34/820	4.15 [2.89–5.75]
				FECT	9/820	1.10 [0.50–2.07]
	Mwape et al.	2013	Zambia	FECT	0/226	0.00 [0.00–1.62*]
				Copro-Ag ELISA	27/226	11.95 [8.02–16.90]
	Thomas	2014	Kenya	Copro-Ag ELISA	278/2003	13.88 [12.39–15.47]
				FECT	4/2059	0.19 [0.05–0.50]
Latin America	Diaz et al.	1992	Peru	microscopy, both directly and after FECT	1/305	0.33 [0.01–1.81]
	Sarti et al.	1992	Mexico	FECT	4/1531	0.26 [0.07–0.67]
	Sarti et al.	1994	Mexico	FECT	2/828	0.24 [0.03–0.87]
	García-Noval et al.	1996	Guatemala	FECT & Copro-Ag ELISA	27/995	2.71 [1.80–3.92]
			Guatemala	FECT& Copro-Ag ELISA	123/713	17.25 [14.55–20.23]
	Sanchez et al.	1998	Honduras	FECT	2/404	0.50 [0.06–1.78]
	Sanchez et al.	1999	Honduras	FECT	12/480	2.50 [1.30–4.33]
	Sarti et al.	2000	Mexico	Copro-Ag ELISA	16/1865	0.86 [0.49–1.39]
				FECT	11/1865	0.59 [0.29–1.05]
	Gomes et al.	2002	Brazil	Copro-Ag ELISA	26/577	4.51 [2.96–6.53]
	Rodriguez et al.	2003	Ecuador	FECT	30/1935	1.55 [1.05–2.21]
				PCR-RFLP (identification)	8/29	27.59 [12.73–47.24]
	García et al.	2003	Peru	microscopy, both directly and after FECT	8/1317	0.61 [0.26–1.19]
				Copro-Ag ELISA	45/1619	2.78 [2.03–3.70]
	Lescano et al.	2009	Peru	FECT	11/898	1.22 [0.61–2.18]
	Praet et al.	2010	Ecuador	FECT	0/674	0.00 [0.00–0.55*]
				MSFT	0/674	0.00 [0.00–0.55*]
	Rodriguez et al.	2006	Ecuador	FECT	14/958	1.46 [0.80–2.44]
				PCR-RFLP (identification)	12/12	100 [73.54–100*]
Asia	Erhart et al.	2002	Viet Nam	FECT	0/210	0.00 [0.00–1.74*]
	Somers et al.	2006	Viet Nam	Copro-Ag ELISA	1/297	0.34 [0.01–1.86]
				KATO	1/297	0.34 [0.01–1.86]
				Worm expulsion & PCR-RFLP for identification	1/297	0.34 [0.01–1.86]
			Viet Nam	Copro-Ag ELISA	3/166	1.81 [0.37–5.19]
				KATO	2/166	1.20 [0.15–4.28]
				Worm expulsion and PCR-RFLP for identification	1/166	0.60 [0.01–3.31]
	Raghava et al.	2010	India	Copro-Ag ELISA	22/729	3.02 [1.90–4.53]
	Jayaraman et al.	2011	India	Copro-Ag ELISA	6/729	0.82 [0.30–1.78]
	Conlan et al.	2012	Laos	FECT and self-report	110/1306	8.42 [6.97–10.06]

Legend: FECT: Formalin-ether concentration technique; KATO: Kato-Katz technique; MSFT: Magnesium sulphate flotation technique; PCR-RFLP: Polymerase chain reaction-restriction fragment length polymorphism; ELISA: Enzyme-Linked Immunosorbent Assay; Copro-Ag ELISA: Coproantigen ELISA; EITB: Enzyme-linked Immunoelectrotransfer Blot; Dem. Rep. of Congo: Democratic Republic of Congo.

^a^: Fecal samples were provided voluntarily from participants when not specified otherwise

*: One sided 97.5% confidence interval

**: Technique applied in fecal samples from seropositive subjects for *T*. *solium* antibodies or circulating antigens

Coprology†: Technique not defined

### Risk factors for human cysticercosis

Twenty-three articles out of the 37 selected articles reported statistically significant risk factors for the presence of *T*. *solium* circulating antigens or *T*. *solium* antibodies. The identified risk factors for the presence of *T*. *solium* circulating antigens in Africa were: insufficient latrines or sanitary toilets [35], washing hands by dipping method [67], history of taeniasis or proximity to tapeworm carriers [37;39], being male [36] and increased risk with age [18;32;35–37;39], while the identified risk factors for presence of anti *T*. *solium* antibodies were: insufficient latrines or sanitary toilets, and increased risk with age [35]. In Latin America the only identified risk factor for presence of *T*. *solium* circulating antigens was associated with increased age [60], while the identified risk factors for presence of *T*. *solium* antibodies were: insufficient latrines or sanitary toilets [45;53;59], lack of potable water [45], poor personal hygiene [41], deficient house hygiene [41], earthen floor [45], presence of infected pigs [48;52], pig owning [45;52;53;59], pork consumption [41;59], history of taeniasis/proximity to tapeworms carriers [41;43;51;52;58], being female [43;46;59], age (risk increased with age) [16;40;42;57;60], increased risk at younger age [54], low education level [45;54], presence of a sewage system [57]. In Asia the only risk factor for the presence of *T*. *solium* circulating antigens was the ownership of pigs [62].

### Meta-analysis

The random effects model used in the meta-analysis gave an overall estimated prevalence for circulating *T*. *solium* antigens in Africa of 7.30% (95% CI [4.23–12.31]) from 9 studies. The overall estimated prevalence for circulating *T*. *solium* antigens in Latin America was 4.08% (95% CI [2.77–5.95]) from 5 studies and 3.98% (95% CI [2.81–5.61]) for Asia from 5 studies. For the anti *T*. *solium* antibodies seroprevalence, the overall estimated seroprevalence in Africa was 17.37% (95%CI [3.33–56.20]) from 2 studies, while in Latin America it was 13.03% (95% CI [9.95–16.88]) from 20 studies and in Asia 15.68% (95% CI [10.25–23.24]) from 2 studies. The prevalence of circulating *T*. *solium* antigens was not significantly different between continents and neither was the anti *T*. *solium* antibodies seroprevalence. The prevalence of circulating *T*. *solium* antigens was significantly lower than the *T*. *solium* antibodies seroprevalence in Latin America and Asia but not in Africa.

## Discussion

The results of this review allowed us to characterize and compare estimates of active infections with *T*. *solium* metacestodes and exposure to the parasite in endemic communities of different countries in three continents. The estimated seroprevalence of anti *T*. *solium* antibodies in Africa (17.37%), Latin America (13.03%) and Asia (15.68%) was not significantly different, which could be interpreted as a similar exposure to *T*. *solium* eggs in these regions, though, within each continent a visible heterogeneity was observed, with seroprevalence for antibodies ranging from 1.82% to 40% as shown in Tables 1, 2 and 3.

The estimated prevalence of *T*. *solium* circulating antigens was higher in endemic areas of Africa (7.30%) compared to Latin America and Asia (4.08% and 3.98%, respectively), though this difference was not statistically significant. When studied in detail, all the African studies but one showed a higher prevalence of active infections than the studies in Latin America and Asia: the prevalence of circulating antigens reported in Latin America and Asia ranged from 0.57 to 9.12%, while the prevalence in Africa ranged from 6.13 to 21.63%, excluding one study from West Cameroon in 2003 reporting a prevalence of 0.68% [32]. The figures observed in Tables 1, 2 and 3 demonstrate big variations in prevalence of active infection with *T*. *solium* cysticerci, similar as observed for sero-prevalence of antibodies.

Studies from the Democratic Republic of Congo, Zambia and Tanzania [18;36;39] revealed very high prevalence figures of circulating antigens not registered in any other part of the world (21.63%, 12.49% and 16.75%, respectively). These figures suggest a higher occurrence of HCC in Africa when compared to Latin America and Asia. The estimated prevalence of *T*. *solium* circulating antigens in each continent was lower than the estimated seroprevalence for *T*. *solium* antibodies in the same continent, but this difference was only statistically significant for Latin America and Asia and not for Africa.

In studies that carried out both antigen and antibody detection tests [16;18;56;60;65] a significantly higher seroprevalence of *T*. *solium* antibodies was observed (Tables 1, 2 and 3) except for one study performed in Senegal [35] in which the number of positive cases for Ag-ELISA and the number of EITB positive cases were similar. However, in that study only a fraction of the positive cases was positive in both tests. This marked difference in the prevalence of circulating antigens and of antibodies has already been observed by Praet et al. (2010) [17]: exposure expressed by antibody seroprevalence could be interpreted as the result of a past infection, current infection or the result of a failed infection, while circulating antigens can only be detected if viable cysticerci are present.

Findings in this review from sero-epidemiological studies have shown that *T*. *solium* transmission varied from one geographical location to another, which raised several questions on the causes of these differences. It is reasonable to think that the variations in the figures observed in this study could be the result of a combination of more than one element capable of affecting the presence of *T*. *solium* cysticercosis in a zone, such as individual host characteristics, parasite singularities and environmental properties each of them prone to change depending on the geographical situation. Table 5 summarizes the potential factors contributing to the variations observed in serology from endemic communities.

**Table 5:** Factors affecting serological variations of human cysticercosis infection and exposure to *T*. *solium* eggs.

	Human host (Accidental intermediate host)	Parasite (*Taenia solium*)	Environment
Factors affecting exposure in a population	Age (time exposed to the parasite)[32]		Number of adult parasites present (tapeworm carriers, hotspots) [58;68]
(Presence of detectable antibodies)	Gender (Role played in different cultures)		Number of eggs dispersed in the environment [69;70]
	Occupation		Egg survival (egg viability) on climatic conditions: Temperature, Humidity, seasonality[69;70]
			Hygiene, sanitation, behavioral practices, agricultural & cooking practices[41]
			Area (endemic)
Factors affecting active infections in a population	Age (Immunosenescence) [60]	*T*. *solium* genotype: Asian or African/Latin American) [71–73]	Egg survival (egg viability) on climatic conditions: Temperature, Humidity, seasonality[69;70]
(Presence of circulating antigens)	Gender (Hormonal profile)[74]	Presence of other *Taenia* species (within host) [63;75]	Frequency and Intensity of exposure [68]
	Ethnicity (Immune characteristics) [10;74;76]		Presence of other *Taenia* species (cross-resistance) [63;75]:
	Nutritional status [37]		Presence of other pathogens [37;77;78]
	Acquired immunity [79]		
	Innate immunity [16;62;72]		
	Immune profile [79;80]		
	Level of exposure		
	Presence of concurrent infections [37;77;78]		

In this review, age was reported as a significant risk factor both for the presence of circulating antigens and/or antibodies, studied reports are compatible with what is described in Table 5. The effect of gender on prevalence is less clear: the studies in Latin America [43;46;59] indicated female to be more likely to have antibodies, while in Africa, in one study, male were more likely to present circulating antigens [36]. The effect of gender on transmission should be further studied to determine whether there is a real difference in physiological gender susceptibility to *T*. *solium* infections [79], or rather a difference associated with the role gender plays in every culture, resulting in different exposure. It is clear that immunity plays a huge role in the host susceptibility/resistance to the parasite but the underlying mechanisms are not well understood. In some cases, the origin of the type of immune resistance/susceptibility present in a population is characterized by the surrounding environment more than of an intrinsic factor in the host. This is the case for low immunity related to the poor nutritive status, which could be a consequence of poverty [37].

Surprisingly, only for the studies from Latin America included in this review, the presence of pigs and the consumption of pork were identified as important risk factors for exposure. In one study from Asia this risk factor was associated with the presence of circulating antigens. In contrast, none on the African studies revealed an association between pig ownership and pork consumption and infection or exposure. These results are compatible with the results from Mwape et al. (2015) [81], reporting other risk factors as the number of inhabitants and age as determinants when studying and ranking *T*. *solium* infections determinants by classification trees. In fact, HCC is the result of the ingestion of eggs coming from tapeworm carriers that could have acquired the adult tapeworm from pork meat either locally produced or transported from other endemic regions [67], thus, presence of pigs in this case is not as relevant for HCC transmission than the local prevalence of tapeworm carriers. HCC is considered a “clustered” disease; some studies have pointed out the importance of the presence of tapeworm carriers in a household triggering HCC in endemic and non-endemic communities[82;83]. Twenty three out of 24 studies reporting taeniasis identified at least one tapeworm carrier, however the diversity of methods used to diagnose taeniasis did not allow to make adequate comparisons between studies, even more, parasitological tests are considered having a low sensitivity, suggesting underestimation of the prevalences. Nevertheless, tapeworm carrier identification can be an important tool to recognize endemicity, in a zone, to identify the sources of infection and to support serological results.

Other important risk factors reported from Africa and Latin America were related to poor sanitary conditions, the deficient handling of human feces, the presence or history of adult tapeworms near the positive cases and low educational level. In the latter case, educational level could be interpreted also as an indicator of rural environment and/or poor household income in endemic communities. An interesting finding is the association of the presence of sewage installations in the house and positivity to EITB in a village in northern Peru [57]. Usually, sewage systems could be interpreted as a sanitary improvement, but in the Peruvian study, sewage installations were present only in some houses, which could result in the concentrated use of hygienic systems, and if they are not handled properly, they can turn the house into a “hotspot” for HCC [58].

Little is known about *T*. *solium* eggs survival in the environment. Some studies have been done on related cestodes like *Taenia saginata* and *Echinococcus* spp. [69], however, extrapolating knowledge from those studies could result in inadequate interpretations because of biological differences between these species. In addition, flies may play a role in the transmission of eggs of some *Taenia* spp. [84;85]; in the case of *T*. *solium*, however, flies were found not to be important because of the coprophagic behavior of pigs that left not enough time after defecation to allow flies to get impregnated with *T*. *solium* eggs[86]. Dung beetles were also shown to carry infective *T*. *solium* eggs in their bodies making them capable of transmitting the parasite [87;88]; however, ingestion of the beetles is needed for effective transmission of the parasite, which makes this route of transmission more likely for porcine cysticercosis than for HCC. Nevertheless, from the experiences acquired in these studies [69; 84–88], it is clear that climatic factors have a direct impact on the viability of taeniids eggs. Thus climatic factors can have a direct influence on the variations observed in the prevalence of active infections and exposure to *T*. *solium* in different climatic settings.

The results observed in the studies performing both Ag-ELISA and EITB could be interpreted as indicating a higher occurrence of active infection in African communities, under a similar exposure to the parasite, suggesting that in this continent there are factors enhancing the human infection regardless of the level of exposure. At a population level, variations and similarities in exposure could be the result of the environmental or behavioral conditions enhancing or disfavoring egg dispersal and survival. The presence of circulating antigens at a population level is the outcome of viable cysticerci, which reflects successful establishment of infection after exposure to *T*. *solium* eggs. This would indicate exposure to infectious eggs and human behavior ensuring the uptake of eggs. Another factor triggering infection is the contact with a large number of eggs, increasing the probability of breaking the immune barrier raising the chance of the cysticerci to establish. A high prevalence of active infections could be as well an indicator of weak immune responses due to multiple factors as described in Table 5. Also, the level of active infections in a population could be inversely proportional to the level of intrinsic factors in the human hosts permitting the presence of innate or acquired resistance to the parasite.

To have a complete picture on the human-to-human transmission for *T*. *solium* cysticercosis, more longitudinal studies should be carried out following strict protocols in order to make them comparable. In the present review, only three studies were found that contributed incidence data, two from the Latin American region [16;50] and one from the African region [18]. The results from Ecuador and Zambia [16; 18] reported at least one change between positivity and negativity in their EITB for *T*. *solium* antibodies in 20–32% of the population studied at one point during a year. For the circulating antigen detection, the percentage of subjects showing a similar phenomenon was about 11.5% of the studied population in Zambia. These results suggest that the number of subjects that are actually exposed to *T*. *solium* eggs or are infected with cysticerci every year is higher than what is reported in prevalence studies. Incidence data could help to explain the interactions of the different components for transmission and their evolution over time. Data obtained from these studies contribute enormous knowledge on how seroprevalence estimation is affected because of the dynamic nature of the infection and of the presence of transient antibodies and possibly transient antigens. Studying the fluctuations of the humoral response over time can also provide information about the level of individual susceptibility by measuring the establishment rate of cysticerci following infection [89].The information obtained from longitudinal studies can also help deciding on the duration of control programs to effectively have an impact in the reduction on the prevalence of the disease.

When designing control programs and strategies, the correct interpretation of exposure patterns can show which factors affect the egg dispersal in the environment and to locate the potential “hotspots”[58;82;83]. By doing so, it will provide the necessary information to create targeted interventions to avoid the spread of the disease taking into account that these patterns vary from one location to another [82;83]. Interpreting the different infection patterns observed in every community will help to prioritize which strategy should be better adapted to the demo-geographical setting in order to decrease the number of new negative health outcomes.

Variations in exposure to the parasite and active infection with *T*. *solium* cysticerci in endemic communities present a challenge when implementing control programs. For these reasons, more studies searching in parallel the presence of antibodies directed against *T*. *solium* metacestodes and the presence of circulating antigens need to be conducted in other endemic communities around the world with detailed characteristics of potential risk factors. It is also recommended that these studies be paired with the study of the adult *T*. *solium* prevalence that can provide another element of the transmission of the parasite.

The main limitation of this study was the lack of a standardized protocol in community-based studies, which made comparable data scarce [90;91]. Moreover, the reliance on only one serological test (EITB or Ag-ELISA) in most of the current sero-epidemiological studies gives only a partial view of the current status of *T*. *solium* infection/exposure in the communities in which they were carried out. Another limitation is that B158/B60 Ag-ELISA and HP10 Ag-ELISA have not been compared in human samples, which could lead to different results given the fact that the monoclonal antibodies used are directed against different antigens in each test. On the other hand, the large time period that elapsed (22 years from 1989 to 2014) between the oldest and the most recent studies may also imply a certain bias in the health, sanitary and educational conditions that may have changed and may have had a direct impact on the *T*. *solium* life cycle, thus affecting its transmission dynamics and prevalence. Another important drawback is the fact that not all the studies selected for this review reported the presence of adult tapeworms. Additionally, the methods used in the studies reporting the presence of tapeworms were diverse and considered with a low sensitivity and specificity, complicating comparisons between studies. Moreover, not all the studies presented details of the studied population such as representativeness of each age group, health status or the presence of other pathogens that could have interfered when characterizing and comparing prevalence figures.

In conclusion, this review demonstrated the variability on the occurrence of active *T*. *solium* infections and exposure to the parasite in endemic zones with some African communities reporting the highest prevalence levels for cysticercosis active infection around the world with results bordering 20%. Several significant risk factors were listed for both active *T*. *solium* infections and exposure to the parasite, some of them being determinant depending on the geographical location, climatic, economic and socio-cultural conditions. The findings in this review should be taken into account in order to help defining priority areas for intervention and control of *T*. *solium*.

## Supporting Information

S1 Checklist Prisma checklist. (DOC)
